# Early career insight

**DOI:** 10.1038/s41390-024-03034-5

**Published:** 2024-01-31

**Authors:** Paris C. Papagianis

**Affiliations:** Department of Pharmacology, School of Medicine, Nursing and Health Sciences, Biomedicine Discovery Institute, Monash University, Clayton, VIC Australia


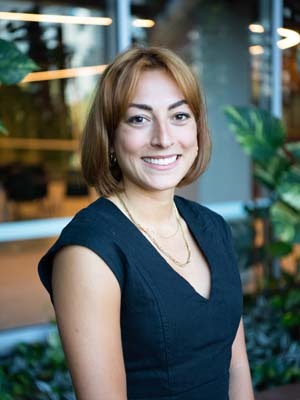


I was born in Melbourne, Australia. I grew up living above my parent’s candy store (where I had unlimited access to sweets) and was known to customers because I routinely interrupted service to ask questions about their life or the latest news headlines. I still ask too many questions, so it is not surprising that I pursued a career in research.

I completed a Bachelor of Science with Honors at Monash University, Australia. I remember learning about fetal development during my undergraduate degree. I was fascinated by the intricate processes involved and astonished that these processes did not go wrong more often! This was my first motivation to get involved in research.

In 2019, I was awarded a joint PhD from Monash University and the University of Western Australia (UWA). I had fantastic mentors and supervisors, Professor Jane Pillow (UWA), A/Professor Tim Moss (Monash) and Professor Graeme Polglase (Monash), who fed my curiosity and encouraged my question-asking. During this time, I was also influenced by my uncle and aunt, who had identical twin boys who were born prematurely. Their twins spent several weeks in intensive care. Now, seeing these boys happy and healthy continues to provide me with motivation to conduct pediatric research.

My PhD supervisor, Professor Jane Pillow, introduced me to highly translational research projects, which I believed could impact the care of prematurely born babies. The research we very recently published in *Pediatric Research*^1^ investigates the use of tapered low-dose dexamethasone to reduce ventilator dependence in premature birth care—still a hotly debated topic in clinical settings.

I now work as a Postdoctoral Research Fellow under the mentorship of A/Professor Jane Bourke at Monash University. My research focuses on lung health and disease spanning early life and into adulthood. My particular interest in lung research stems from the critical importance of lung health over the lifespan—the lungs are where life begins and where it fails at end of life.

There are many other mentors who have inspired me throughout my short career, including Prof Claudia Nold, Prof Marcel Nold, Prof Alan Jobe, Prof Mary Wlodek and Prof Donna Geddes, Prof David Walker and Dr Bernard Thebaud (to name a few!)—I suppose the message is surround yourself with bright minds and good role models!

**Your advice to those coming along behind you**:

Firstly, find yourself a mentor outside of your research area! This has been one of the best things for my professional development. An unbiased opinion on how to move forward to achieve career goals, objectives or advice on submitting grants is invaluable.

Secondly, change it up! I have studied many different things in my short time as an ECI. I have changed the organs or disease setting I study, the cell types I am interested in, the lab groups I work with. I recently started a project on gut development and necrotizing enterocolitis that has challenged me, but has been incredibly rewarding! Importantly this feeds back into my other work and provides novel approaches to tackling diseases of prematurity through exposure to different technologies, lab groups and expertise.
